# Scalp Metastasis from Leiomyosarcoma of the Inferior Vena Cava Sign as the First Clinical Sign: A Case Report

**DOI:** 10.1155/2012/631010

**Published:** 2012-10-10

**Authors:** Isabel Prieto Muñoz, Jose Pardo Masferrer

**Affiliations:** ^1^Radiation Oncology Department, Capio Fundacion Jimenez Diaz University Hospital Avenida Reyes Católicos 2, 28040 Madrid, Spain; ^2^Radiation Oncology Department, Son Espases University Hospital, Carretera de Valldemossa 79, 07010 Palma de Mallorca, Islas Baleares, Spain

## Abstract

The presentation of scalp metastases from leiomyosarcoma of the vena cava is an extremely infrequent event. There are no other publications that describe such finding and very few of leiomyosarcoma in vessels. About this event we have reviewed the English literature describing studies on scalp metastases and skin metastases in general: their incidence, origin, clinical appearance, meaning, and diagnosis. The case we describe would be the second one presented worldwide because, as far as we know, it has been only one more published in 2005.

## 1. Introduction

Leiomyosarcoma of the inferior vena cava is a rare neoplasm with poor prognosis. It accounts for only about 0.5% of adult soft tissue sarcomas, although within the group of sarcomas arising from the venous system, it is the most common location, nearly 50% of cases [1].

Leiomyosarcoma is known for its resistance to chemotherapy, radiation therapy, and propensity for haematological dissemination. The diagnosis used to be delayed due to the absence of symptoms during months. Most of them are metastatic disease at the moment of the histological confirmation. The presentation of scalp metastases from leiomyosarcoma of the vena cava is an extremely infrequent event. 

## 2. Case Report

A 29-year-old woman was referred with a subcutaneous nodule in the scalp of three-month duration as first symptom. The nodule was 1 cm sized, not ulcerated, and without swelling signs. It did not show hair loss and had grown asymptomatically.

**Figure 1:**
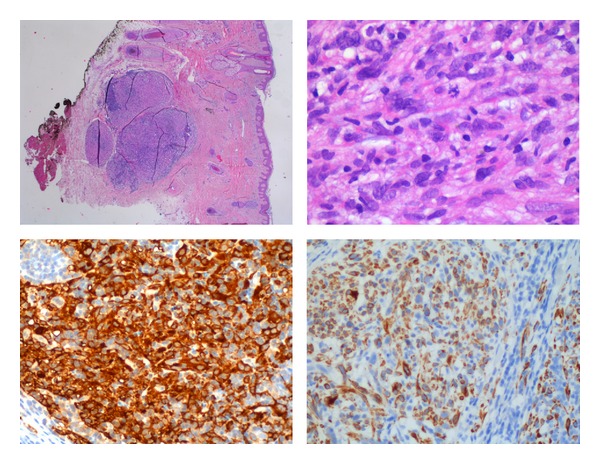
Tumor cells from scalp stained with hematoxylin-eosin, caldesmon, and desmin.

The first biopsy revealed an epithelioid sarcoma (mesenchymal neoplasm with intense nuclear atypia, pleomorphism, and moderated mitotic activity with areas with solid patterns of growing and tumoral necrosis). Tumoral cells expressed vimentin immunoreactivity and epithelial membrane antigen (EMA). They did not express any specific mesenchymal marker. High Ki67 expression level was observed (>25%). As long as margins were involved, the second biopsy—complete resection—revealed a sarcoma with muscle differentiation, a leiomyosarcoma grade II. The immunohistochemical results expressed vimentin, desmin, caldesmon, and EMA (Figure 1). Other markers were negative (actine, cytokeratins, CD 34, p53 protein).

Computed tomography (CT) revealed a mass (diameter 3.6 cm) near from the right adrenal gland that included inferior vena cava with heterogeneous capture of contrast and fat density. Magnetic resonance image demonstrated that the heterogeneous mass was in this vessel and had low-density centrum and probable necrosis. The mass displaced the right adrenal gland. It was showed one nodular lesion in liver in segment VIII that seemed to be a metastasis as first possibility. Tomography with emission of positrons (PET) confirmed the mass near from the right adrenal gland with malignance signs (maximum standardized uptake value was 3.8) and the focal deposit in liver. Percutaneous needle biopsy of the mass confirmed the diagnosis.

She received neoadjuvant chemotherapy with Taxotere (100 mg/m^2^ day 8) and gemcitabine (900 mg/m^2^ days 1 and 8) each 21 days. After 3 cycles, it was found a partial response of the retroperitoneal mass without changes in the liver. Three months after diagnosis, surgery was planned. The mass and the vena cava involved were resected en bloc and an endoluminal prosthesis was emplaced. The tumor sized 4.5 cm and invaded the 50% of the vessel lumen. It compressed the adrenal gland without infiltration. The hepatic deposit was also resected (1.6 cm). After this procedure, she received three cycles more with the same scheme. 

After 6 months, TC showed liver and lungs relapse. She was given a second-line chemotherapy with ifosfamide (2500 mg/m^2^ days 1–4) and adriamycin (30 mg/m^2^ days 1–3), 6 cycles. After 6 months enlargement of the pulmonary nodes were observed and a new hepatic metastasis was found. She received a new scheme with trabectedin (1.3 mg/m^2^), and after 12 cycles, it was stopped because of hepatic progression. She died 59 months from diagnosis.

## 3. Discussion

Cutaneous metastases of internal malignances seem to occur infrequently, although medical publications report an incidence rate up to 10.4% excluding cutaneous infiltrations of haematological malignances [2–5]. These metastases may be the first indication of the clinically silent visceral malignances. The search for an unknown primary tumor is a real request. The regional distribution, although not always predictable, is related to the location of the primary malignancy and the mechanism of metastatic spread [3].

Cutaneous metastases are perceived as a sign of advanced disease and are regarded as a grave prognostic indicator. It usually signifies a widespread systemic disease and high mortality rate.

The incidence of skin metastases varies among different studies [3, 6, 7]. Generally, these metastases differ in their origin in males and females. In the former, they usually arise from carcinomas of the lungs, the colon, and the oral cavity and malignant melanomas. If melanoma is excluded, the lung cancer is the most frequent source of cutaneous metastases in men [4]. In females, the most frequent sources of the skin metastases are carcinomas of breast (most common primary origin), colon, ovaries, and lungs as well as melanomas. Other studies include renal cell, uterine cervix [8, 9], and gastrointestinal cancer [10–12]. The pattern of metastasis also depends on the age of the patient [6]. Sarcomas seldom metastasize to the skin [3]. 

In most cases, skin metastases arise long time after the initial diagnosis of the primary malignancy. Occasionally, they may be detected simultaneously with or even before the diagnosis of a primary tumor [3]. 

Without specific assignment of cutaneous metastases to different organs, metastases are mainly located on the chest (30.3%), abdomen (20.2%), and scalp (12.6%) [7]. Other series include head and neck (28%), the trunk (40%), the extremities (18%), and multiple sites that include scalp location (14%) [3, 7].

Positron-emission tomography (PET) examinations with 8–53% detection rates play an important role for diagnosis, but they reveal a high number of incorrect positive findings (sensitivity of 91.9% and specificity of 81.9%) [2]. Currently, the gold standard remains conventional histological analysis combined with immunohistochemical staining. Nashan et al. [2] and Hussein [3] describe the possible mechanisms of the skin metastases and the difficulties for the analysis of the biopsy. The vast majority of these metastases are confined to the dermis and/or subcutaneous fatty tissue. Therefore, tumor cells can grow either in a nodular or star-like pattern, within or around dilated lymphatic and blood vessels, or in small groups in a linear arrangement dissecting collagen bundles. The connective tissue involved may appear relatively normal or fibrotic. Only sometimes, the cutaneous spread reaches the epidermis and invades it. The absence of epidermal involvement is indicative that the tumor is metastatic [3–5].

The clinical presentations of the skin metastases vary over a wide morphologic spectrum, although most frequently are multiple nodules [2–4]. They that occur by lymphatic or hematogenous spread induce nodular, inflammatory, and telangiectatic skin lesions. Carcinomas may also invade the skin by direct extension and produce pagetoid infiltration of the epidermis along with dermal plaques. Some metastases, particularly when they are a single lesion, can closely simulate a benign cyst, keratoacanthoma, basal cell carcinoma, melanoma, or nodular fasciitis. Nashan et al. and Hussein and describe these wide varieties of clinical presentations [2, 3]. 

In most cases, the metastatic deposit shows histological features that resemble the underlying primary malignance. However, metastases may be more anaplastic and exhibit less differentiation. Immune markers and ultrastructural studies are needed [2, 4, 5, 7].

There is a paucity of literature in regards to leiomyosarcoma of the vena cava with metastases to the scalp. Vandergriff et al. [13] have reported in the English-language literature from 1960 only 15 cases of leiomyosarcoma with metastases to the skin. The uterus is the overall most common site of origin metastasizing to the skin. Moreover, primary tumors of the genitourinary system account for more than half of the cases. Other sites of primary tumors include the gastrointestinal tract, the heart, the breast, and the retroperitoneum. The author describes one case from vascular structures, from the femoral vein. There are to our knowledge no other cases from these structures: this one that was published by Gow in 2005 [13] and our case that we describe in this paper.

Vandergriff concludes that the scalp is the most common cutaneous site of involvement of metastatic leiomyosarcoma occurring in half of all patients with skin metastasis. It would be necessary another 50 years more in order to report at least 15 new additional cases.
